# Sympathetic Arousal during a Touch-Based Healing Ritual Predicts Increased Well-Being

**DOI:** 10.1155/2015/641704

**Published:** 2015-07-05

**Authors:** Karin Meissner, Anne Koch

**Affiliations:** ^1^Institute of Medical Psychology, Ludwig-Maximilians-University of Munich, 80539 Munich, Germany; ^2^Institute for the Study of Religion, Ludwig-Maximilians-University of Munich, 80539 Munich, Germany

## Abstract

*Objective*. There is mounting evidence that more elaborate treatment rituals trigger larger nonspecific effects. The reasons for this remain unclear. In a pilot field study, we investigated the role of psychophysiological changes during a touch-based healing ritual for improvements in subjective well-being. *Methods*. Heart rate, respiratory rate, and skin conductance levels (SCL) were continuously assessed in 22 subjects before, during, and after a touch-based healing ritual. Participants rated their expectations and subjective well-being was assessed before and after the ritual by the “Short Questionnaire on Current Disposition”. *Results*. Subjective well-being increased significantly from before to after the ritual. The analysis of psychophysiological changes revealed a significant increase in respiratory rate from baseline to ritual, while skin conductance, heart rate, and heart rate variability did not change. Increases in SCL as well as decreases in respiratory rate from baseline to ritual were significantly associated with improvements in subjective well-being. Regression analyses showed increases in SCL to be the only significant predictor of improvements in well-being. *Conclusion*. Higher sympathetic arousal during a touch-based healing ritual predicted improvements in subjective well-being. Results suggest the occurrence of an anticipatory stress response, that is, a state of enhanced sympathetic activity that is known to precede relaxation.

## 1. Introduction

Intervention effects consist of both specific and nonspecific effects [1]. Nonspecific effects (synonymous with placebo effects) are thought to be due to psychobiological processes triggered by the overall therapeutic context [2] and are mediated by the patient's expectations towards the treatment, previous experience with treatments (learning), and characteristics of the patient-provider-interaction [3]. Characteristics of treatments and treatment settings can further modulate the response [4]. For example, in a meta-analysis on migraine prophylaxis, sham acupuncture and sham surgery were associated with significantly larger responder ratios than oral placebo pills [5]. This suggests that more complex treatment rituals are associated with larger nonspecific effects.

Many treatments from complementary and alternative medicine (CAM) use elaborate, complex healing rituals and may therefore provide a good opportunity to study nonspecific effects. One example is ritual touch healing, in which a therapist “transfers energy” to the diseased sites of the patient and/or “removes blockages from the patient's energy field” [6]. Participation in these carefully performed, long-lasting rituals may give rise to intensified experiences, which in turn may enhance the patient's expectations towards the power of the ritual. The slight stimulation of the skin by slow, stroking movements of the healer's hand may additionally trigger nonspecific treatment effects by enhancing trust and bond between the healee and the healer: pleasant touch has rewarding properties and can set in motion an affective/motivational response, including emotional arousal and the release of the bonding-hormone oxytocin [7].

Therefore, a promising approach to learn more about the neurobiological mechanisms underlying the effects of touch-based healing rituals may be to investigate psychophysiological changes during the ritual and to relate these changes to subjective benefit thereafter. Psychophysiological changes are closely related to changes in emotional arousal [8] and can therefore provide an insight into otherwise “invisible” processes during a healing ritual. So far, few studies have measured psychophysiological parameters during touch-based healing rituals and existing results are contradicting. Wardell and Engebretson [8], for example, reported changes of systolic blood pressure, electromyography, and skin temperature in the direction of relaxation during a Reiki session in healthy volunteers. In contrast, Engle and Graney [9] found evidence for increased sympathetic tone (vasoconstriction) during Therapeutic Touch (TT) in a healthy cohort. Finally, Cox and Hayes [10] measured heart rate, arterial blood pressure, respiration, and peripheral oxygen saturation before, during, and after TT in critical-care patients and found no significant changes in any of the physiological variables. The investigators of these studies had expected a relaxation response already during the touch-based healing ritual to occur, and findings in the opposite direction were classified as “harmful” [9]. However, according to recent research findings, an initial sympathetic activation precedes a state of relaxation [11]: within this so-called “anticipatory stress response,” an individual is proposed to evaluate whether the situation is safe before bonding and relaxation processes may emerge.

In this pilot study, we investigated subjective and psychophysiological changes during a standardized touch-based healing ritual that aimed to reduce stress and increase general well-being. In contrast to former studies, we hypothesized that the touch-based healing ritual would increase autonomic arousal during the ritual and that this arousal would be positively associated with improvements in subjective well-being thereafter.

## 2. Methods

### 2.1. Participants

Twenty-two participants were recruited (11 participants with and 11 participants without previous experience with the healing ritual). Given the pilot character of the study, a convenience sample was used with no restrictions regarding age, sex, diseases, or medication. Participants gave their informed consent to participate in the study.

### 2.2. Procedure

Participants were instructed not to eat or to drink in the two hours before testing. Upon arrival, participants received information about the experimental procedure and informed consent was obtained. Before and after the ritual, participants filled in the questionnaire on current disposition and a questionnaire to assess demographic variables as well as expectations towards the ritual (see below). Just before the ritual, the electrodes to measure heart rate, respiratory activity, and skin conductance were attached and participants were instructed to find a comfortable position on a stool in the middle of the ceremony room. Before (“baseline,” 5 min), during (“ritual,” about 10 min), and after the ritual (“after ritual,” 5 min), psychophysiological activities were continuously assessed. After detaching the electrodes, participants underwent in-depth interviews with one of the authors (Anne Koch) in a separate room (results have been reported separately [10]).

### 2.3. Healing Ritual

The touch-based healing ritual of the White Eagle Lodge is located in the hybrid tradition of theosophical-spiritist-Christian chakra work. For study purposes, a female, experienced healer performed a standardized ritual aimed at reducing stress and at improving general well-being. The healee was sitting on a stool in the ceremony room, facing an altar with ritual symbols, such as an illuminated cross in a circle, and ritual utensils, such as a lighted candle, since the healing energy is conceived of as light flooding through the healee. The healer started the ritual treatment beginning at the “crown chakra” on top of the head of the healee and worked her way down through the seven chakras to the “root chakra” located at the height of the pubic bone, thereby mostly not touching the healee but stroking along the body surface at a little distance of about two inches. The healer only touched and stroked slightly the shoulders and the back line of the vertebra and let her hands slide down simultaneously on the belly and the back at the height of the solar plexus. The healer started and closed the session with prayers suggesting drawing the attention away from the “outer senses” to the “inner world” and calling for the help of healing spirits.

### 2.4. Assessment of Subjective Well-Being and Expectations

Subjective well-being was assessed before and after the ritual by using the “Short Questionnaire on Current Disposition” (SQD) [12]. This questionnaire uses six bipolar items (tense, calm; apprehensive, unperturbed; worried, unconcerned; anxious, relaxed; skeptical, trusting; uneasy, comfortable) and allows measuring subjective well-being within short test-retest intervals.

In order to assess expectations towards the ritual, we asked participants the following question before the ritual: “What do you personally expect from the touch healing ritual you will receive?” Answer options were “cure,” “clear improvement,” “slight improvement,” “no improvement,” and “do not know” (adapted from [13]). For further analyses, the answers to this question were dichotomized (participants responding with one of three highest options versus remaining participants).

### 2.5. Physiological Recordings

Electrocardiogram, electrodermal activity, and respiratory activity were continuously recorded during the experimental session by using a BIOPAC MP 150 device (BIOPAC Systems Inc., Goleta, CA, USA) with AcqKnowledge 3.7.2 software for data acquisition. The electrocardiogram was sampled at 500 Hz. The electrodermal and respiratory signals were digitized at a rate of 15.625 Hz.

Electrodermal activity was measured by two disposable Ag/AgCl electrodes (Cleartrace, Conmed, Utica, NY, USA) placed at the thenar and hypothenar of the nondominant hand and connected to the BIOPAC amplifier module GSR100C. The signal was filtered by an analog 1 Hz first-order low pass filter.

Respiratory activity was measured by a strain gauge transducer (TSD201, BIOPAC Systems Inc., Goleta, CA, USA) attached around the thorax and connected to the BIOPAC amplifier module RSP100C.

### 2.6. Data Reduction and Analysis

Cardiac interbeat intervals were extracted from the ECG signal as the intervals between successive R peaks by using the peak detection function implemented in AcqKnowledge 3.7.2. Cardiac interbeat intervals were examined and manually screened for artifacts according to the procedure developed by Porges and Byrne [14]. The cardiac intervals were averaged for the baseline, ritual, and postritual periods and were then converted into heart rate (HR) for the ease of reading.

Positive and negative peaks of each respiratory cycle were extracted by using the peak detection function implemented in AcqKnowledge 3.7.2. The intervals between positive peaks were used to estimate respiratory periods [15]. Respiratory periods were averaged for the baseline, ritual, and posttest periods and were finally converted into respiratory rate (RR) for the ease of reading.

Electrodermal activity signals were averaged for each experimental condition. The square root (sqrt) of skin conductance levels (SCL) was taken from each mean to obtain normal distributions [16].

The root mean square of successive differences (RMSSD) was calculated from the cardiac interbeat interval time series [17] of each experimental session. The RMSSD is viewed as a time-domain-based index corresponding to parasympathetic neural regulation of the heart [18] and was used to estimate heart rate variability.

### 2.7. Statistical Analysis

SQB scores were analyzed using repeated-measures analyses of variance (ANOVA) with “condition” (baseline, after ritual) as within-subject factor. Possible differences in SQB changes according to previous experience with the ritual (yes/no) and expectations (positive/indifferent) were explored by using students *t*-tests.

Psychophysiological outcomes (HR, RR, RMSSD, and SCL) were analyzed using repeated-measures analyses of variance (ANOVA) with “condition” (baseline, ritual, and after ritual) as within-subject factor. Significant main effects for “condition” were followed up by Bonferroni-corrected post hoc tests.

Pearson's correlations were used to investigate the relationships between changes in subjective well-being and changes in psychophysiological outcomes from baseline to ritual. To identify possible predictors for improvements in SQD scores, a stepwise linear regression analysis with changes in SQD scores as the dependent variable and age as well as psychophysiological changes from baseline to ritual as the independent variables was performed.

For all statistical tests, a significance level of *p* < 0.05 was assumed.

## 3. Results

### 3.1. Characteristics of the Study Sample

Table 1 summarizes the characteristics of the 22 participants. Half of the participants had previous experience with the touch-based healing ritual of the White Eagle Lodge for reasons that ranged from somatic and psychological to karmic ones (e.g., chronic back pain, relief from stress and tension, easing the burden of self-responsibility and/or care for elderly parents, and connecting with energies and spirits from the spiritual world). Eight participants took pharmacological drugs (4 antihypertensives, 2 psychopharmacological drugs, and 2 thyroid hormones).

### 3.2. Expectations

Thirteen participants expected the ritual to be effective (“positive expectations”), while nine participants did not know (“indifferent expectations”) (Table 1). Positive expectations were more frequent in the experienced group: while ten out of eleven participants with previous experience in the healing ritual reported positive expectations, eight out of eleven ritual-naïve participants reported indifferent expectations (Chi-Quadrat = 9.2, *p* = 0.008).

### 3.3. Changes in Well-Being

**Figure 1:**
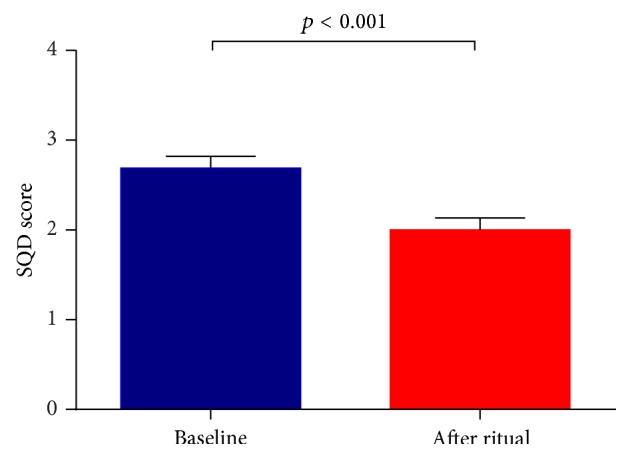
Increase in well-being from before to after the ritual, as assessed by the “Short Questionnaire on Current Disposition” (SQD) (lower scores indicate greater well-being). Error bars indicate standard error.

Scores in SQD decreased significantly from baseline to after ritual, indicating an improvement in subjective well-being (Figure 1; Table 2). Decreases in SQD scores did not differ between participants with or without previous experience with the ritual (*p* = 0.888) or between participants with positive or indifferent expectations towards the ritual (*p* = 0.278).

### 3.4. Changes in Psychophysiological Variables

The repeated-measure ANOVA for RR showed a main effect for condition, which was due to a significant increase of RR from baseline to ritual (Bonferroni-corrected *p* = 0.001, Table 2). The main effect of condition for HR showed a trend for significance (*p* = 0.088), with decreases of HR during the ritual and increases thereafter (Table 2). RMSSD and SCL did not change significantly during the ritual (Table 2).

Since psychophysiological changes may be affected by age, sex, and medication status, sensitivity analyses were performed by means of *t*-tests. Participants younger than 50 years (*n* = 10) showed larger increases in RR as well as larger decreases in HR from baseline to ritual than elder participants (RR: 2.9 ± 1.1 SD versus 0.8 ± 1.6 SD, *p* = 0.005; HR: −3.6 ± 3.7 SD versus 0.7 ± 1.7 SD, *p* = 0.014). Respective changes in SCL and RMSSD did not differ between age groups (data not shown). Neither sex nor medication status affected any of the psychophysiological parameter changes from baseline to ritual (data not shown).

### 3.5. Relationships between Changes in Well-Being and Psychophysiological Variables

Table 3 summarizes the results of the correlational analyses. Decreases in SQD scores were associated with increases in SCL from baseline to ritual (*r* = −0.61, Bonferroni-corrected *p* = 0.036) as well as with decreases in RR from baseline to ritual (*r* = 0.64, Bonferroni-corrected *p* = 0.006). No such associations were found for HR and RMSSD (*r* = −0.06 and *r* = −0.12, resp.).

Stepwise linear regression analysis on changes in SQD scores with changes of SCL, RR, HR, RMSSD, and age group included as independent variables revealed relative increases in SCL to be the only independent predictor for improved well-being (Table 3).

## 4. Discussion

Subjective well-being increased significantly from baseline to after ritual. The analyses of psychophysiological changes showed significant increases in RR and nonsignificant decreases in HR from baseline to ritual, while RMSSD and SCL did not change. Only increases of SCL from baseline to ritual, however, predicted improvements in subjective well-being thereafter.

Ritual touch healing interventions, such as Reiki and TT, have been reported to affect the activity of the autonomic nervous system in shifting autonomic activity from sympathetic to parasympathetic dominance, indicating relaxation [8, 19, 20]. For example, after completion of a 30-minute Reiki session patients were more relaxed than before, as indexed by decreases in systolic blood pressure and neck muscle tension [8]. Our study differed from these studies by focusing on psychophysiological changes* during* a touch-based healing ritual and relating these changes to subjective improvement thereafter. This approach revealed that participants with a larger increase in SCL during the ritual showed larger improvements in well-being, as indexed by larger decreases of SQD scores from before to after the ritual.

Electrodermal activity is widely used by emotion researchers and is generally considered a good predictor of emotional arousal, as it is not under voluntary control and is highly sensitive to sympathetic nervous system changes [21, 22]. Emotional arousal is observed during both positive and negative emotional states and, in addition to increased SCL and SCR (i.e., skin conductance responses to a defined event), is accompanied by increases in RR and decreases in HR [23]. Increases in RR and (nonsignificant) decreases in HR occurred during the ritual period of our study (Table 2). The lack of a significant increase of SCL from baseline to ritual in our study is most probably due to the fact that SCL levels tend to decrease during prolonged periods of bodily rest [24]. Thus, the initially high SCL during the baseline measurement presumably blurred moderate increases of SCL during the ritual period. Taken together, our data indicate the occurrence of slight emotional arousal during the ritual period.

Increases of sympathetic activity have been shown to precede relaxation responses. Within this so-called “anticipatory stress response,” an individual is proposed to evaluate whether the situation is safe before bonding and relaxation processes may emerge. The finding that only changes in SCL but not RR, RMSSD, or HR predicted changes in subjective well-being in our study is in line with prior findings that the anticipatory stress response is associated with increases in norepinephrine levels and enhanced peripheral vasoconstriction, both markers of sympathetic activity [11]. In contrast to RR, RMSSD, and HR, SCL is regarded a pure sympathetic measure [24].

The results of this pilot study should be regarded preliminary. We investigated a small convenience sample without applying exclusion criteria. Although our sensitivity analyses suggest no effect of medication status or sex on our results, we cannot completely exclude this possibility. We included participants with and without previous experience with the ritual but did not match them according to age, sex, and medication. Thus, conclusions with regard to possible differences between these groups are premature.

In conclusion, our results indicate that sympathetic arousal during a touch-based healing ritual predicted improvement in well-being thereafter. The state of enhanced sympathetic activity during the ritual has been interpreted as an anticipatory stress response that is known to precede relaxation. The results of this pilot study are promising and justify larger, well-controlled studies in order to better understand the working mechanisms of complex healing rituals. This is important since complex healing rituals get more and more popular in Western societies, which could not least be due to the large nonspecific effects they induce.

## Figures and Tables

**Table 1:** Baseline characteristics.

Characteristics	*N* (%)
Female/male	14 (64)/8 (36)
Age	
20–40	8 (36)
40–60	4 (18)
≥60	10 (46)
Previous experience with the ritual, yes/no	11 (50)/11 (50)
Expectation towards the ritual	
Very effective	2 (9%)
Slightly effective	7 (32%)
Effective	4 (18%)
Not effective	0 (0%)
Do not know	9 (41%)

**Table 2:** Behavioral and psychophysiological variables before, during, and after the ritual.

	Baseline	Ritual	After ritual	ANOVA	
	Mean (SD)	Mean (SD)	Mean (SD)	*F*_cond_	*p* value
SQD (score)	2.7 (0.6)	—	2.0 (0.6)	28.2	**<0.001**
Heart rate (min^−1^)	74.5 (9.7)	73.6 (9.3)	75.0 (9.6)	2.6	0.088
RMSSD (ms)	25.2 (13.6)	26.2 (16.2)	23.4 (13.5)	0.5	0.526
Skin conductance level (sqrt *μ*mho)	3.4 (1.3)	3.2 (1.6)	3.3 (1.7)	0.6	0.489
Respiratory rate (min^−1^)	12.3 (3.9)	13.8 (4.4)	13.3 (4.9)	4.0	**0.039**

SQD: Short Questionnaire on Current Disposition; RMSSD: root mean square of successive differences.

**Table 3:** Stepwise regression analysis for psychophysiological variables (changes from baseline to ritual) and age on changes in SQB scores.

Predictor variables	*r* ^2^	*B*	*t*	*p* value
Dependent: ΔSQB (score)				
Model	0.38			
ΔSCL (sqrt *μ*mho)		−0.619	−3.154	**0.006**
Excluded variables				
ΔRR (min^−1^)		0.344	1.628	0.124
ΔHR (min^−1^)		0.102	0.508	0.619
ΔRMSSD		−0.155	−0.752	0.464
Age (<50/≥50 years)		−0.037	−0.175	0.864

SQD: Short Questionnaire on Current Disposition; SCL: skin conductance level; RR: respiratory rate; HR: heart rate; RMSSD: root mean square of successive differences.
